# Do differences in profiling criteria bias performance measurements? Economic profiling of medical clinics under the Korea National Health Insurance program: An observational study using claims data

**DOI:** 10.1186/1472-6963-11-189

**Published:** 2011-08-16

**Authors:** Hee-Chung Kang, Jae-Seok Hong

**Affiliations:** 1 Health Insurance Review & Assessment Institute, Health Insurance Review & Assessment Service, Seoul, Republic of Korea

**Keywords:** cost efficiency, economic profiling, National Health Insurance

## Abstract

**Background:**

With a greater emphasis on cost containment in many health care systems, it has become common to evaluate each physician's relative resource use. This study explored the major factors that influence the economic performance rankings of medical clinics in the Korea National Health Insurance (NHI) program by assessing the consistency between cost-efficiency indices constructed using different profiling criteria.

**Methods:**

Data on medical care benefit costs for outpatient care at medical clinics nationwide were collected from the NHI claims database. We calculated eight types of cost-efficiency index with different profiling criteria for each medical clinic and investigated the agreement between the decile rankings of each index pair using the weighted kappa statistic.

**Results:**

The exclusion of pharmacy cost lowered agreement between rankings to the lowest level, and differences in case-mix classification also lowered agreement considerably.

**Conclusions:**

A medical clinic may be identified as either cost-efficient or cost-inefficient, even when using the same index, depending on the profiling criteria applied. Whether a country has a single insurance or a multiple-insurer system, it is very important to have standardized profiling criteria for the consolidated management of health care costs.

## Background

With a greater emphasis on cost containment in many health care systems, it has become common to evaluate each physician's relative resource use [1-6]. The motivation for this economic profiling is primarily financial in that physicians identified as being inefficient are considered to be wasting health plan resources and these physicians can be encouraged to change their practice pattern [7].

Previous studies have shown that differences in health status among patients may influence treatment costs within defined episode types and that the health care costs of treated patients may differ significantly among physicians [5-8]. Efforts to identify physician outliers who show extreme practice patterns and to encourage them to change their behaviors have become important for the management of health care costs [8,9]. The Korea National Health Insurance (NHI) program detects such outliers using economic profiles.

The O/E ratio, which compares the observed cost (O) with the expected cost (E), is the measure typically used for provider economic profiling [5,10]. In this article, we refer to this score as cost efficiency; however, health economists have objected to this usage because they have long used *efficiency *to refer to the cost of resources used in achieving a given outcome or benefit to the patient. In September 2005, a meeting convened by the Ambulatory Quality Alliance and the National Committee for Quality Alliance determined that *cost efficiency *was an acceptable term for the relative use measure and that, because it does not control for quality or patient benefit, it must be distinguished from *efficiency*, which controls for outcomes [11]. Thus, a cost-inefficient physician here means one who claims more costs than expected.

$$\text{Cost}-\text{efficiency Index}=\frac{Observed\cos t}{Expected\cos t}=\text{O}∕\text{E ratio}$$

However, even the same index may render different values depending on profiling criteria, including the data used and risk-adjustment methods. For example, if pharmacy costs are not included when profiling costs or different case-mix classification systems are applied, this may affect a provider's calculated cost efficiency, which could in turn change his/her performance rating.

Within a single NHI program, multiple monitoring systems may use the same performance measures but apply different profiling criteria. This could make it difficult to achieve cost efficiency in the NHI system.

The present study explored the major factors that influence the economic performance rankings of medical clinics in the Korea NHI program by assessing the consistency between cost-efficiency indices constructed using different profiling criteria.

## Methods

### 1. Study setting

The NHI program of Korea covers the whole population as a compulsory social insurance system. The National Health Insurance Corporation (NHIC) is the sole insurer and is responsible for operating the program; however, the Health Insurance Review Agency (HIRA) is in charge of reviewing providers' claims, and the Ministry of Health and Welfare (MOHW) supervises the program as a whole. Its main sources of funding are contributions from the insured and government subsidies. Medical care benefit services include diagnoses, tests, drugs, medical materials, treatments, surgeries, preventative care, rehabilitation, hospitalization, nursing, and transportation. Basically, the medical care benefit costs are reimbursed through a fee-for-service system for all services and provider levels [12].

Patients pay certain portions of treatment costs as co-payments. The co-payments for outpatient care vary depending on the total medical charges as well as the level of health care facility. Inpatient care requires patients to pay 20% of the total medical charges.

A patient can select any practitioner or any medical care institution, but to be treated at a secondary hospital (specialized general hospitals), one must present a referral slip issued by the doctor who saw the patient first.

Medical care institutions are classified as follows, based on the number of beds: clinics (fewer than 30 beds), hospitals (30-99 beds), and general hospitals (more than 99 beds); all of these can provide outpatient services [12].

This study was limited to medical clinics, excluding those that provide eastern medicine or dental services. Medical clinics were considered to be appropriate for studying physician-profiling methods because 98% (25,168) of all clinics (25,789) were sole practices as of 2006 [13].

The reimbursement process starts with medical care institutions' submitting claims for their medical services to HIRA for review. In this fee-for-service reimbursement system, this review is aimed at minimizing the risk of paying for excessive or unnecessary patient care and also encouraging more efficient care through profiling and feedback systems (HIRA, 2008). NHIC makes the payment to the medical care institution based on the results of the review [14].

The HIRA has operated two systems to monitor the practice patterns of medical clinics: the Comprehensive Management for Appropriate Medical Services System (CM System) and the Notice System for Autonomous Corrective Action (Notice System). The CM System is basically a function of HIRA's consulting services to medical clinics, and their advice is not legally binding. However, the Notice System is an operation delegated to the HIRA by the MOHW, and refusal to take autonomous corrective action after receiving a notice may lead to an onsite investigation under the anti-fraud enforcement program. A medical clinic identified to have made false claims in an onsite investigation will face an administrative penalty. If the wrongdoing is serious, criminal charges may also be filed against the institution [14].

Both systems use the O/E ratio to calculate cost efficiency, but profiling criteria suggested for the two systems are different. The Notice System was developed in 1986, and no established patient classification system existed at that time. Instead, the system classified patients with the three-digit code of the Korean Classification of Disease(KCD) developed on the basis of the International Classification of Diseases-Tenth Edition (ICD-10), and subdivided into 2 groups depending on whether the patient had a surgical operation or not. Medical clinics claimed pharmacy costs as well as professional costs until the separation of prescribing and dispensing medicine was implemented in 2000; since then, medical clinics have claimed professional costs only, and the cost-efficiency index of the Notice System has been bases solely on professional costs [15].

Within the CM System, which was introduced in 2003, the Korean Outpatient Group (KOPG) system is used for case-mix classification, and each clinic's cost-efficiency index is calculated based on medical care benefit costs, which include professional costs and drug costs. This is aimed at controlling pharmaceutical costs by placing responsibility for prescription drug costs on medical clinics and limiting their prescriptions.

The KOPG system was developed by the HIRA in 2003, with reference to the American Ambulatory Patient Groups, version 2.0, taking into account the clinical characteristics of ambulatory patients and the similarity of their resource use [16,17]. In this system, all claims are classified according to significant outpatient procedures or therapies. Those claims with significant outpatient procedures are divided into 172 groups according to patient age. The other claims are divided into 262 groups according to patient age using subcategories of the KCD or are divided into five error-KOPG groups and 43 ancillary-only-KOPG groups by ancillary tests or procedures [17].

Although these two systems were developed at different times, they have a similar process and objective: to profile medical care institutions on their relative costs and to inform those medical care institutions of the outcomes, thereby encouraging them to change their practice patterns. However, because the NHI program has been operating the two systems simultaneously since 2003, some medical clinics have received contradictory information from the two systems and have complained about the reliability of that information. Accordingly, increasing attention has been paid to the main factors that reduce the level of agreement between the indices of the two systems.

### 2. Study data and measures

Study data on medical care benefit costs for outpatient care at nationwide medical clinics were collected from the claims database which HIRA reviewed in April 2007. We excluded 13 specialty clinics, such as pneumonolgy, clinical pathology, and anatomic pathology, because each specialty had fewer than 10 clinics across Korea. Study subjects finally recruited were 23,112 medical clinics and 22,088,649 patients who visited these clinics. The specialty classification of a medical clinic with more than one practicing physician was based on the chief physician's specialty. The number of clinics and their patients by specialty are presented in Table 1. The proportion of pharmacy costs against total costs was, on average, 29.76%. The lowest proportion of pharmacy costs was shown in diagnostic radiology (10.61%), and the highest portion in internal medicine (38.23%). The correlation coefficient between medical care benefit costs including pharmacy costs and those excluding pharmacy costs was lowest in internal medicine (0.87). However, as the mean coefficient (0.95) shows, it was high overall.

**Table 1:** Number of clinics and their patients, proportion of pharmacy costs, and correlation between total costs including and excluding pharmacy costs by specialty

Specialty	No. of clinics	No. of patients	% of total cost represented by pharmacy cost (mean ± sd)	Correlation between costs including and excluding pharmacy costs^1)^
General clinics	4,695	3,807,917	32.04 ± 23.84	0.99
Internal medicine	3,611	4,852,887	38.23 ± 25.23	0.87
Pediatrics	2,128	2,333,778	26.18 ± 19.80	0.99
Otorhinolaryngology	1,737	2,561,254	24.10 ± 15.40	0.99
Orthopedic surgery	1,725	1,585,045	20.23 ± 21.56	0.99
Obstetrics & gynecology	1,604	873,524	21.09 ± 20.82	0.98
Ophthalmology	1,207	1,515,235	23.38 ± 17.31	0.97
General surgery	1,193	794,618	27.65 ± 23.32	0.97
Family medicine	1,055	1,009,541	34.11 ± 23.20	0.95
Urology	888	581,503	30.82 ± 23.46	0.90
Dermatology	803	779,600	29.02 ± 21.96	0.93
Psychiatry	708	308,835	19.00 ± 27.05	0.99
Anesthesiology	588	296,653	28.90 ± 25.95	0.99
Neurosurgery	362	298,801	27.49 ± 25.29	0.99
Rehabilitation medicine	276	158,182	27.17 ± 26.74	0.99
Diagnostic radiology	244	156,622	10.61 ± 21.37	0.96
Neurology	132	104,304	41.56 ± 28.60	0.92
Thoracic surgery	82	64,099	31.97 ± 23.83	0.97
Plastic surgery	74	6,251	13.22 ± 20.15	0.98

Total	23,112	22,088,649	29.76 ± 23.80	0.95

1) Pearson's correlation coefficient

The cost-efficiency index is termed the costliness index (CI) in the CM System, whereas it is called the Y Index in the Notice System. In this study, however, the general term cost-efficiency index was used because this study focused on evaluating the agreement between cost-efficiency indices with different profiling criteria. We created eight types of cost-efficiency index by cross-substituting different profiling criteria suggested in the Notice System and the CM System (Table 2).

**Table 2:** Cost-efficiency indices and their profiling criteria

Cost-efficiency (CI) indices		Case-mix classification		Data range		Cost range	
		
		KCD & surgery experience	KOPG	300 most frequent disease cases	All cases	Excluding pharmacy costs	Including pharmacy costs
CI_1	KOPG, all cases, including pharmacy costs		Y		Y		Y

CI_2	KOPG, all cases, excluding pharmacy costs		Y		Y	Y	

CI_3	KOPG, 300 most frequent disease cases, including pharmacy costs		Y	Y			Y

CI_4	KOPG, 300 most frequent disease cases, excluding pharmacy costs		Y	Y		Y	

CI_5	KCD & surgery, all cases, including pharmacy costs	Y			Y		Y

CI_6	KCD & surgery, all cases, excluding pharmacy costs	Y			Y	Y	

CI_7	KCD & surgery, 300 most frequent disease cases, including pharmacy costs	Y		Y			Y

CI_8	KCD & surgery, 300 most frequent disease cases, excluding pharmacy costs	Y		Y		Y	

The formula for calculating the cost-efficiency index is as follows:

the h clinic's cost-efficiency index,

$$CI_{h}=\frac{Observed\;\cos t}{Expected\;\cos t}=\frac{\overset{n}{\underset{g=1}{\sum }}C_{hg}\times N_{hg}}{\overset{n}{\underset{g=1}{\sum }}C_{g}\times N_{hg}}$$

h: clinic,

g: by case-mix classification (KOPG or KCD & surgery),

N_hg_: the number of cases (patients) by case-mix classification in the h clinic,

C_g_: average costs by case-mix classification in clinics with the same specialty as the h clinic,

C_hg_: average costs by case-mix classification in the h clinic

**Figure 1:**
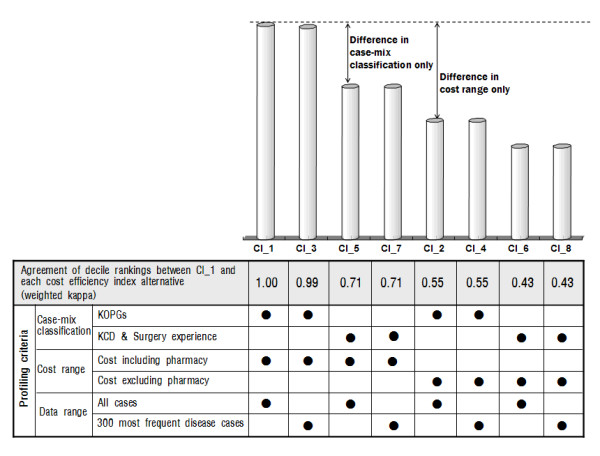
**Agreement in decile rankings between the baseline cost-efficiency index (CI_1) and each cost-efficiency index alternative assessed using the weighted kappa statistic**.

The CM System and Notice System have different case-mix classification, data range, and cost range in profiling criteria. The Notice System's case-mix classification categorizes a patient with a three-digit code based on the primary diagnosis (KCD) and his or her surgery experience, whereas the CM System follows the system developed by the KOPG. In terms of data range, the Notice System calculates each clinic's efficiency index based solely on the patients who visited with one of the 300 frequent disease groups of the same specialty clinics; this accounts for more than 90% of all cases. However, the CM System includes all cases. With regard to cost range, pharmacy costs are excluded from total costs in the Notice System but included in the CM System. In Table 2 and Figure 1, CI_1 is the same as the CI in the CM System, and CI_8 is the same as the Y Index in the Notice System.

### 3. Statistical analyses

We calculated eight types of cost-efficiency index for ambulatory care costs at a medical clinic and investigated the agreement between index rankings to explore the main factors that influence their agreement. The agreement between indices at high and low outlier thresholds of 10% was explored, and then the agreement between decile rankings of the two indices was assessed using the weighted kappa statistic proposed by Landis and Koch [18]. If the ranges of two indices agree completely, the kappa statistic is near or equal to 1; if they lack agreement, the kappa statistic is near or equal to 0. When a kappa statistic is greater than 0.8, the agreement between indices can be interpreted as a very good or almost perfect agreement. Also, agreement in the range of 0.61-0.80 is interpreted as good or substantial agreement, whereas 0.41-60 is moderate agreement, 0.21-0.40 is fair agreement, and less than 0.20 is poor agreement. Generally, if kappa is less than 0.4, the agreement is interpreted as not good [18,19]. Analyses were conducting using SAS (ver. 9.1).

## Results

### 1. High outlier agreement and low outlier agreement

When we investigated the agreement at the high outlier threshold of 10% between CI_1 and each cost-efficiency index alternative, the highest rate of agreement (99.9%) was observed in the pair of CI_1 and CI_3, which differed in data ranges. In the pair of CI_1 and CI_5, which differed in case-mix classifications, the rate of agreement fell to 68.6%. It was reduced further to 57.5% in the pair of CI_1 and CI_2, which differed in cost ranges. The lowest level of high outlier agreement was shown in the pair of CI_1 and CI_6 and that of CI_1 and CI_8, where agreement was 44.7% and 44.8%, respectively. CI_1 and CI_6 differed from each other in case-mix classification, data range, and cost range, whereas CI_1 and CI_8 differed in case-mix classification and cost range. However, in all pairs, little variation in agreement was due to a difference in data range. The low outlier agreement also showed the same trend as the high outlier agreement in each pair of cost-efficiency index alternatives (Table 3).

**Table 3:** Agreement at high and low outlier thresholds of 10%: number of clinics by alternative cost-efficiency indices and their percentage relative to the baseline cost-efficiency index

	CI_1	CI_2	CI_3	CI_4	CI_5	CI_6	CI_7	CI_8
high outlier 10%	2,311 (100.0)	1,328 (57.5)	2,308 (99.9)	1,328 (57.5)	1,585 (68.6)	1,033 (44.7)	1,585 (68.6)	1,035 (44.8)

low outlier 10%	2,310 (100.0)	1,317 (57.0)	2,308 (99.9)	1,316 (57.0)	1,685 (72.9)	1,033 (44.7)	1,681 (72.7)	1,030 (44.6)

### 2. Agreement between decile rankings

When CI_1, the baseline cost-efficiency index, was compared with each cost-efficiency index alternative to assess the agreement between the decile rankings using the weighted kappa statistic, the highest level of agreement was observed in CI_3 (k = 0.99), which differed in data range compared with CI_1 (Figure 1). The lowest level of agreement was shown in CI_6 and CI_8 (k = 0.43), which differed from CI_1 in case-mix classification and cost range. CI_6 and CI_8 differed in data range. The pair of CI_1 and CI_8 demonstrates how the profiling criteria of the CM System and the Notice System differ (k = 0.43). For the pair of CI_1 and CI_2 in terms of total costs, including and excluding pharmacy costs, the weighted kappa statistic was 0.55, whereas in the pair of CI_1 and CI_5, with different case-mix classifications, it was 0.71. Thus, the inclusion or exclusion of pharmacy costs was shown to have a greater effect on agreement than a difference in case-mix classification had.

The agreement of decile rankings between CI_1 and each cost-efficiency index alternative was further analyzed according to physician specialty (Table 4). In the pair of CI_1 and CI_2, with regard to total costs including and excluding pharmacy costs, internal medicine showed the lowest agreement of decile rankings (k = 0.37). However, surgical specialties, such as orthopedic surgery, anesthesiology, neurosurgery, rehabilitation medicine, and diagnostic radiology, had a statistically high level of agreement. Variations in agreement due to a difference in case-mix classification were smaller than were those due to pharmacy costs. However, in the case of case-mix classifications, medical specialties showed relatively high agreement.

**Table 4:** Agreement in decile rankings between the baseline cost-efficiency index and each cost-efficiency index alternative by specialty, assessed using the weighted kappa statistic

Specialty	CI_1	CI_2	CI_3	CI_4	CI_5	CI_6	CI_7	CI_8
General clinics		0.49	0.99	0.49	0.64	0.33	0.63	0.33
Internal medicine		0.37	1.00	0.37	0.80	0.33	0.80	0.33
Pediatrics		0.65	1.00	0.65	0.90	0.62	0.89	0.62
Otorhinolaryngology		0.61	1.00	0.61	0.55	0.36	0.55	0.36
Orthopedic surgery		0.72	1.00	0.72	0.70	0.56	0.70	0.56
Obstetrics & gynecology		0.64	1.00	0.64	0.80	0.58	0.80	0.57
Ophthalmology		0.56	1.00	0.56	0.80	0.52	0.80	0.52
General surgery		0.56	1.00	0.56	0.65	0.40	0.65	0.39
Urology		0.40	1.00	0.40	0.76	0.37	0.76	0.37
Dermatology		0.41	1.00	0.41	0.77	0.40	0.77	0.40
Family medicine		0.45	1.00	0.45	0.72	0.34	0.71	0.34
Psychiatry		0.67	1.00	0.67	0.62	0.51	0.62	0.51
Anesthesiology		0.75	1.00	0.75	0.65	0.52	0.65	0.52
Neurosurgery		0.70	1.00	0.70	0.64	0.47	0.64	0.47
Rehabilitation medicine		0.83	1.00	0.83	0.66	0.56	0.66	0.56
Diagnostic radiology		0.79	1.00	0.79	0.82	0.71	0.81	0.68
Neurology		0.43	1.00	0.43	0.62	0.31	0.62	0.32
Plastic surgery		0.83	1.00	0.83	0.68	0.63	0.68	0.63
Thoracic surgery		0.64	1.00	0.64	0.54	0.37	0.54	0.38

## Discussion

In medical clinics under the Korea NHI program, the cost-efficiency index calculated using all cases and that calculated using only those cases that present with one of the 300 most frequent disease groups were barely different. The greatest difference between cost-efficiency indices resulted from differential policies with regard to pharmacy costs. When the cost-efficiency index for total costs including pharmacy costs was compared with the index for total costs excluding pharmacy cost, the agreement between the two indices was only 55%. The agreement between indices was also reduced, to 71%, when a difference in case-mix classification was involved. However, case-mix classification was less influential than was cost range. Among all pairs of cost-efficiency indices, the lowest level of agreement (43%) was observed between CI_1 and CI_8. This pair differed in three criteria: data range, cost range, and case-mix classification. These results suggest that the application of different profiling criteria to similar indices may result in contradictory outcomes that may be confusing to a medical institution and may impair the reliability of provider-profiling systems. Thus, the standardization of profiling criteria among provider feedback systems is important for achieving more efficient spending at the macro level of the insurance program.

Because we cannot know in real terms whether a cost-efficiency index calculated for a health care provider using claims data informs us of correct rankings, we cannot evaluate the validity of the index directly [5]. The reliability of the cost-efficiency index is thus important in terms of its utilization [4]. From the insurance administrator's point of view, the cost-efficiency index for a health care provider is used to identify a physician who has tended to provide higher- or lower-cost services than expected to patients [1]. In the United States, provider-profiling systems were introduced or adopted mostly by managed care insurers who needed to manage insurance finances [2,4,20]. Recently Medicare, a social insurance program administered by the United States government, has also implemented a resource-utilization report plan through which information on cost efficiency is provided to an individual provider as a way of slowing the trend toward increasing health care costs [21].

This result is not consistent with the findings of Thomas (2006), who evaluated the agreement among decile rankings of cost-efficiency indices for total costs including pharmacy costs and excluding pharmacy costs using claims data from physicians who were enrolled in a university-owned mixed-model health maintenance organization in southeastern Michigan, USA, using the weighted kappa statistic [11]. The inclusion or exclusion of pharmacy costs did not greatly affect the rankings. However, Thomas suggested that attention needs to be paid to the effect of excluding pharmacy costs in the case of clinical specialties for which the proportion of pharmacy costs to total costs is relatively high and those in which the correlation between costs including pharmacy costs and those excluding pharmacy costs is relatively low. Our findings confirm the need for such attention.

In the present study, differences among profiling criteria affected cost-efficiency profiling to different degrees depending on specialty and the ratio of pharmacy costs to total costs (Tables 1 and 4).

Two specialties discussed in the study by Thomas (2006) with regard to the effect of excluding pharmacy costs were cardiology and neurology [11]. The percentages of total costs represented by pharmacy costs in these specialties were 28% and 36%, respectively, and the correlation between costs with and without pharmacy costs were 0.975 and 0.903, respectively. In our study, the proportion of pharmacy costs to total costs was, on average, 29.8%, a relatively high level, whereas many specialties had low correlations between costs with and without pharmacy costs. Accordingly, the exclusion of pharmacy costs is understood to have resulted in the underestimation of costs expended by healthcare providers, which greatly lowered the overall agreement between indices (Tables 1 and 4). Additionally, the level of agreement was at its lowest in internal medicine, which was characterized by the highest proportion of pharmacy costs and the lowest correlation between total costs with and without pharmacy costs among specialties. This result suggests that if a provider specialty shows a high proportion of pharmacy costs, we need to take into account whether pharmacy costs were included in total costs when profiling the provider.

The agreement between two index alternatives with a difference in case-mix classification was 71%, indicating that the agreement between indices is also affected by case-mix classification. However, this is still higher than the agreement between two indices for which the pharmacy cost is a factor, and 71% itself is statistically good agreement. Such results indicate that although the accuracy of case-mix adjustment methods also may affect agreement between indices, case-mix adjustment is still very important for the reliability and accuracy of provider profiling [5,22,23]. In the same specialty group, some physicians may treat patients with clinically difficult conditions and may therefore incur higher costs than expected. Thus, case-mix adjustment is also important for equitable physician profiling [7,10,24].

Our study has several advantages over previous ones. First, despite the usefulness of cost-efficiency indices, their reliability has been questioned by some providers. However, few studies have addressed the reliability and accuracy of cost-efficiency indices [4,5,21,23]. We analyzed the agreement between indices with the same formula but different profiling criteria compared to those used in provider-profiling systems under the Korea NHI program.

Second, we attempted to identify important factors that must be taken into account to improve the accuracy and reliability of cost-efficiency indices. Due to a much easier access to a computerized database of claims, provider profiling has increasingly been introduced at various areas and levels of medical institutions under the Korea NHI program. In line with this trend, the likelihood that cost-efficiency indices with the same objective would have different values due to differences in profiling criteria is also increasing. By revealing such factors for consideration, we hope our study provides a useful cautionary message to governments or insurers who are planning the extensive application of physician profiling.

Third, we calculated and analyzed cost-efficiency indices for 23,112 medical clinics that claimed costs from the Korean NHI program in April 2007. These clinics correspond to 90% of the 25,780 medical clinics registered at the Health Insurance Review and Assessment Service of Korea (HIRA) as of the end of 2006 [12]. Thus, our study subjects represented almost all medical clinics operating under the Korean NHI program at that time. As we analyzed almost all medical clinics, our results can be regarded by other countries or healthcare systems as valuable evidence with respect to enhancing agreement between indices

However, the time lag between when services are provided and when they are billed could be a limitation of our study. HIRA builds a database when claims are submitted, and one of the reasons for doing so is to monitor cost efficiency for a medical institution and thereby to provide relevant feedback as soon as possible. If HIRA chooses to monitor cost efficiency using claims data constructed by the incurred time, this will lead to a delay until all claims of that time have been filed. In reality, as the National Health Insurance Act of Korea sets forth, the insurer shall pay for medical care benefit costs without delay upon a medical institution's filing a claim, and most medical institutions submit their claims in a batch every month. In the April 2007 database that we used, 95% of cases occurred within 3 months before the claim, thus minimizing any potential influence of the time lag between services rendered and claim acceptance.

In addition, we took the average costs of medical clinics with the same specialty as each clinic's expected cost. This is a common method when profiling a provider's performance, although the additional costs of caring for more patients or sicker patients cannot be fully adjusted across medical clinics [25]. Multivariate regression models can be used to estimate the expected costs of each provider, using R-square values for a valid comparison of different systems of physician profiling. It should be used for the further study.

If profiling outcomes for a health care provider differ by insurer, the provider is unlikely to trust the profiling bodies, and this could make the consolidated management of costs more difficult. An effort to standardize profiling criteria for indices among similar systems in a universal health insurance system, as in Korea, and among related organizations in a multi-insurers system is thus required. To enhance the accuracy and reliability of physician profiling indices, above all, the standardization of methods of determining cost range and of case-mix adjustment, as presented in this study, must be initiated.

## Conclusion

It is becoming increasingly common to profile health care providers according to their relative resource use, and relevant cost-efficiency indices have been developed accordingly. The objective of a cost-efficiency profiling system is to encourage cost-inefficient healthcare providers to change their practice patterns by providing feedback on their cost efficiency. However, although the same cost-efficiency index is applied, a health care provider may be profiled as either efficient or inefficient, depending on the profiling criteria. Whether a country has universal health insurance, like Korea, or a multiple-insurers system, it is very important to standardize profiling criteria for the reliability and accuracy of cost-efficiency indices and, ultimately, for the consolidated management of healthcare costs. When standardizing profiling criteria, two factors especially must be taken into account: the inclusion or exclusion of pharmacy costs in total costs and the method of case-mix classification.

## Competing interests

The authors declare that they have no competing interests.

## Authors' contributions

HCK conceptualized the study, conducted the data analysis, interpreted results, and drafted the manuscript. JSH substantially contributed to and reviewed the drafts of the manuscript. All authors read and approved the final manuscript.

## Pre-publication history

The pre-publication history for this paper can be accessed here:

http://www.biomedcentral.com/1472-6963/11/189/prepub
